# Effect of Difference of Sensory Modality in Cognitive Task on Postural Control During Quiet Stance

**DOI:** 10.3390/s25041273

**Published:** 2025-02-19

**Authors:** Yusuke Sakaki, Naoya Hasegawa, Ami Kawata, Hiromasa Akagi, Minori Sawada, Hiroki Mani

**Affiliations:** 1 Graduate School of Health Sciences, Hokkaido University, Sapporo 060-0812, Japan; yellow-elephant1005@eis.hokudai.ac.jp (Y.S.); chiroru259@eis.hokudai.ac.jp (A.K.);; 2 Department of Rehabilitation Science, Faculty of Health Sciences, Hokkaido University, Sapporo 060-0812, Japan; 3 Department of Health Sciences, School of Medicine, Hokkaido University, Sapporo 060-0812, Japan; 4 Faculty of Welfare and Health Science, Oita University, Oita 870-1124, Japan; mani-hiroki@oita-u.ac.jp

**Keywords:** auditory cognitive load, dual task, sensory modality, postural control, static balance, visual cognitive load

## Abstract

Cognitive loads impact postural control; however, the specific influence of sensory modalities employed in cognitive tasks during motor-cognitive dual tasks remains unclear. This study investigated the distinct effects of visual and auditory cognitive tasks on static postural control while controlling for differences in task content. Twenty-five healthy young adults were instructed to maintain a quiet stance on a force plate under three cognitive task conditions: a single motor task (control), a paced visual serial addition task (visual), and a paced auditory serial addition task (auditory). Center of pressure (COP) displacements were measured, and both linear (e.g., sway area) and non-linear assessments of postural control were analyzed. Results revealed a significant reduction in sway area during cognitive tasks compared to the control condition. However, under the auditory condition, the power spectrum density of COP displacements in the moderate frequency band was significantly higher than those in the control and visual conditions, accompanied by a notable increase in the mean power frequency. These findings suggest that auditory cognitive load exerts a more significant effect on postural control than visual cognitive load during motor-cognitive dual tasks. This highlights the relevance of sensory modalities in cognitive loads for effective fall-risk assessment.

## 1. Introduction

Each year, one in three community-dwelling older adults experiences at least one fall [1]. Falls lead to persistent deficits in strength and mobility, resulting in loss of independence, reduced quality of life, and an increased risk of recurrent injury [2]. In addition, falls represent a significant burden on the healthcare system, and as the population continues to age, there are concerns that this burden will increase even further [3,4]. Effective fall-prevention interventions are, therefore, urgently needed in order to maintain the quality of life of older adults and to reduce the burden on the healthcare system. Robust evidence from systematic reviews indicates that the loss of postural control is a critical predictor of future falls [5,6]. Maintaining static and dynamic postural stability depends on the interaction of sensory components, including somatosensory, visual, and vestibular inputs [7]. Consequently, traditional postural control assessments often utilize quiet standing tasks with sensory manipulation (e.g., eyes open or closed) [8]. However, activities of daily living involve performing multiple tasks simultaneously, where one task impacts another. Indeed, research has shown that postural control is influenced by cognitive loads [9], and cognitive function correlates with fall risk [10]. Recently, several systematic reviews have highlighted the importance of conducting fall-risk assessments under motor-cognitive dual-task conditions [1,11]. Thus, understanding how cognitive loads influence postural control is essential for developing new fall-prevention strategies.

Previous studies on motor-cognitive dual tasks have reported that performing cognitive tasks during a quiet stance improves postural control in healthy young and older adults, as well as in patients with stroke and multiple sclerosis, as shown through linear and non-linear measurements [12,13,14,15,16]. For instance, linear measurements such as sway area, sway amplitude, and mean variability of the displacements and velocity of the center of pressure (COP) during a quiet stance were found to decrease during cognitive tasks [12,13,14,15,16,17,18]. Conversely, cognitive loads increased COP mean velocity [13,18]. These findings are inconclusive. The former indicates the reduction in postural sway with variability, meaning more energy-efficient and stable postural control [19,20]. Thus, motor-cognitive dual tasks improve postural stability, whereas the latter implies more effortful postural control, meaning reduced stability [8,19]. To address these ambiguities, several studies have employed non-linear measurements, which reflect postural strategies alongside linear measurements. The mean power frequency (MPF) of COP, which characterizes the frequency dynamics of postural sway, is a typical non-linear measurement used to assess postural control [21]. Research has shown that MPF increases with cognitive tasks [12,13,22,23,24]. Some studies associate increased MPF with a shift toward an automatic postural control strategy, which enhances postural stability by diverting attention from postural sway, indicating efficient control [12,13,24,25]. Others suggest that increased MPF corresponds to a shift toward a stiffness strategy involving greater muscular activity to maintain stability, representing inefficient control [22,23,26]. These discrepancies underscore that the effect of increased MPF on postural control remains inconsistent. Frequency analysis further elucidates postural control mechanisms by dividing the COP signal into four frequency bands: ultra-low (under 0.10 Hz), very low (0.10–0.39 Hz), low (0.39–1.56 Hz), and moderate (1.56–6.25 Hz) [27,28,29]. These bands capture the contribution of sensory systems, including visual, vestibular, and proprioceptive inputs, as well as automatic postural strategies. Studies have linked greater contributions from the very low and low frequency bands to shifts toward automatic postural control strategies [29,30], while greater contributions from the moderate band are associated with increased muscular activity, reflecting a stiffness strategy [27,31]. Thus, frequency analysis, combined with MPF, offers detailed insights into postural control mechanisms under dual-task conditions.

Although most cognitive tasks involve visual or auditory information, several studies suggest that the influence of cognitive tasks on postural control varies depending on the sensory modality used [32,33,34,35,36]. This phenomenon has been attributed to differences in the neural pathways involved in processing sensory information. However, the specific influence of different sensory modalities on postural control remains unclear. Some studies report that sway area or sway amplitude decreases more during auditory cognitive tasks than during visual cognitive tasks [32,33], while others report the opposite [35,36]. Importantly, the cognitive tasks used in these studies differ not only in sensory modality but also in task content. For instance, Richer and Lajoie [36] employed a visual cognitive task in which participants viewed three-digit numbers presented consecutively on a monitor and counted the occurrences of a predetermined digit (e.g., 3). Conversely, in the auditory task, participants listened to letters presented sequentially through a speaker and counted occurrences of a predetermined letter (e.g., B). These tasks differed not only in the sensory modality (visual vs. auditory) but also in the type of information presented (digits vs. letters) and its complexity (three-digit sequences vs. single letters). Therefore, the task conditions employed in previous studies do not adequately evaluate how different sensory modalities influence postural control. In addition, previous studies showed a change in postural stability using only linear but not non-linear measurements. If changes in sensory modalities affect not only postural sway but also postural strategies, it is necessary to adapt the sensory modalities used in motor-control dual tasks according to the objectives of the fall-prevention training. To address these issues, it is essential to investigate the effects of sensory modality on postural control while controlling for task content differences.

This study aimed to examine the effects of sensory modality in cognitive tasks on static postural control under conditions that excluded differences in task content by comparing visual and auditory cognitive tasks. Several studies have reported that the degree of cognitive requirements affected the performance of motor-cognitive dual tasks [29,34]. We hypothesized that (1) sway area or sway range decreases during both visual and auditory cognitive tasks, and (2) the static postural control strategy was more affected by the auditory cognitive task compared to the visual one, even when the cognitive task content remained constant.

## 2. Materials and Methods

### 2.1. Participants

We conducted a randomized, single-blind, cross-over design. A total of 25 healthy young adults participated in this study. The inclusion criteria were as follows: (a) no history of neurological or musculoskeletal disorders, and (b) no uncorrected vision or auditory impairments that could affect their ability to complete the testing procedures. All participants provided informed consent, as approved by the Institutional Review Board of the Faculty of Health Sciences at Hokkaido University (No. 23-61). All procedures were conducted in accordance with the Declaration of Helsinki (1964).

### 2.2. Equipment

A force plate (Kistler, Winterthur, Switzerland) was used to measure the COP coordinates in the anteroposterior (AP) and mediolateral (ML) directions. The force plate data were sampled at a frequency of 1000 Hz and filtered using a fourth-order 8 Hz low-pass zero-lag Butterworth filter. Cognitive tasks were presented on a 103-inch screen or through two speakers positioned approximately 3 m from the participants. LabVIEW version 2016 (The National Instruments Corp., Austin, TX, USA) was used to program the cognitive tasks and collect force plate data.

### 2.3. Procedure

**Figure 1:**
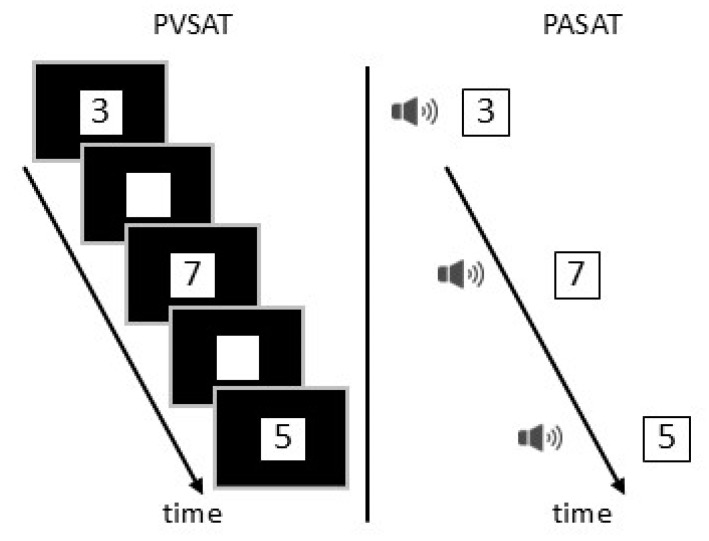
The visual cognitive task (PVSAT) and auditory cognitive task (PASAT) required participants to sum a single-digit number presented every 2 s with the number that preceded it. In the PVSAT, numbers were displayed at the center of the screen, while in the PASAT, numbers were delivered as auditory stimuli through speakers.

Participants were instructed to stand still and minimize sway, keeping their arms at their sides and their feet together for 60 s during each cognitive condition (control, visual, and auditory). In the visual and auditory conditions, participants performed a paced visual serial addition task (PVSAT) and a paced auditory serial addition task (PASAT), respectively, as cognitive tasks [37,38]. Both tasks required participants to add a single-digit number presented every 2 s to the one preceding it. In the PVSAT, numbers were displayed at the center of the screen [37], while in the PASAT, auditory numbers were delivered through speakers [38] (Figure 1). During the control and auditory conditions, participants were instructed to “gaze at a black point placed at the center of the screen”. Additionally, during the visual and auditory conditions, participants were asked to “answer the cognitive task as accurately as possible”.

Participants first completed five trials under the control condition. Following this, they randomly performed either the visual or auditory condition for five consecutive trials, and subsequently switched to the other condition for five trials. To prevent fatigue, participants were given at least 5 min of rest between conditions.

### 2.4. Data Analysis

All signals were processed offline using MATLAB software (version R2019b; MathWorks, Natick, MA, USA). Force plate data were collected for 60 s, with the first and last 5 s removed from the analysis to eliminate the effects of task initiation and completion. The primary outcome was the COP data derived from the force plate. To evaluate postural stability, the 95% confidence area of the COP in the horizontal plane (sway area) was calculated under each condition. Additionally, standard deviation (SD), mean velocity, and amplitude of COP displacements in the AP and ML directions (SD AP, SD ML, Velocity AP, Velocity ML, Range AP, Range ML) were calculated to characterize direction-specific postural control.

To further investigate the effects of cognitive tasks at the frequency level in the AP and ML directions, COP displacements were analyzed using a fast Fourier transform, and the power spectrum density was obtained. The MPF in the AP and ML directions (MPF AP and MPF ML) was calculated from the power spectrum density within the 0.01 Hz to 8 Hz range. Additionally, the power spectrum was divided into four frequency bands in the AP and ML directions: ultra-low (under 0.10 Hz), very low (0.10–0.39 Hz), low (0.39–1.56 Hz), and moderate (1.56–6.25 Hz). Each power spectrum density was expressed as a percentage of the total density spectrum [27,28,29].

In the visual and auditory conditions, the correct response rate for the PVSAT or PASAT was calculated as a measure of cognitive performance.

### 2.5. Statistical Analysis

The sample size was determined to detect a difference in sway area between visual and auditory conditions using the *t*-test model for Bonferroni pairwise comparisons in G*power 3.1. The effect size was estimated from a pilot study conducted with eight participants (calculated effect size of *d* = 0.91). To achieve a statistical power level of 0.95, the required sample size for this study was determined to be *n* = 23. Many of the previous studies investigating the effect of cognitive loads on static postural stability had 10–20 healthy young participants [39]. Therefore, to account for potential dropouts and outliers, the final sample size was increased by 10% (*n* = 25).

The average of each parameter across five trials in each condition was calculated and used for statistical analysis. A logarithmic transformation was applied to all parameters to ensure normal distribution. The distribution was examined using the Shapiro–Wilk test. As normal distribution was ensured on all measurements except cognitive performance, a one-way repeated-measures analysis of variance (rANOVA) was conducted with the cognitive conditions (control, visual, and auditory) as a factor to analyze differences in postural stability measures and power spectrum density. Post hoc analysis was performed using Bonferroni pairwise comparisons. For each significant main effect, the effect size was calculated using eta squared (*η*^2^) for the analysis of variance (ANOVA) models. As data were non-normally distributed, the Wilcoxon signed-rank test was used to compare cognitive performance between visual and auditory conditions. Statistical analysis for all parameters was performed using IBM SPSS statistics (version 27.9; IBM, Armonk, NY, USA), with statistical significance set at *p* < 0.05.

## 3. Results

Due to missing data caused by machine malfunction, one participant was excluded from the study. Additionally, one participant was excluded for declining to perform the cognitive tasks. The final sample size consisted of 23 healthy young adult participants (12 males and 11 females, age: 22.3 ± 1.6 years; height: 163.6 ± 8.5 cm; weight: 56.0 ± 8.1 kg).

### 3.1. Postural Stability Measurements

**Figure 2:**
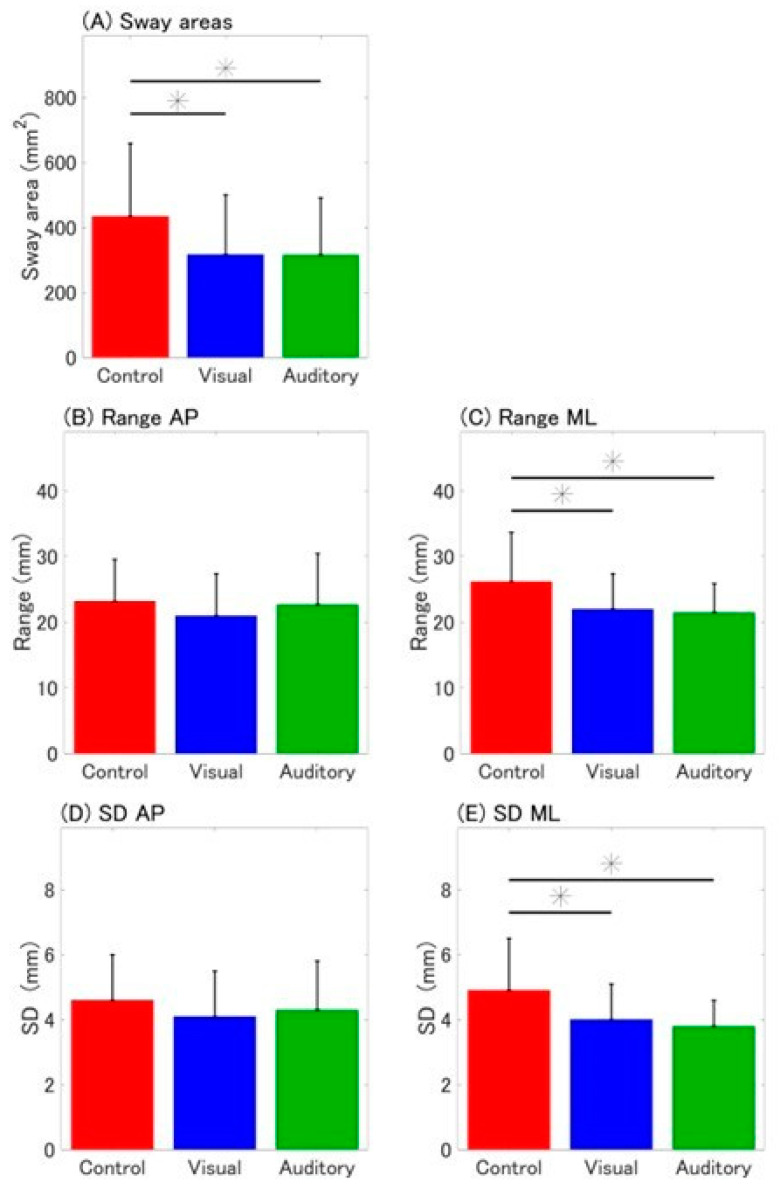
Bar plot of postural stability measures under each cognitive condition. Mean and standard deviation (SD) of (**A**) sway areas, (**B**,**C**) range in the anteroposterior (AP) and mediolateral (ML) directions, and (**D**,**E**) SD of COP displacement in the AP and ML directions are presented. Error bars indicate standard deviations. * *p* < 0.05.

A significant main effect of cognitive conditions was observed for the sway area, Range ML, and SD ML (Table 1). Participants demonstrated significantly smaller sway areas under both visual and auditory conditions compared to the control condition (*p* = 0.011 and *p* = 0.007, respectively; Figure 2A). However, no significant difference was observed between the visual and auditory conditions (*p* = 1.000). Similarly, Range ML and SD ML were significantly lower under the visual and auditory conditions compared to the control condition (Range ML: *p* = 0.001 and *p* < 0.001; SD ML: *p* = 0.001 and *p* < 0.001, respectively; Figure 2C,E). Again, no significant difference was found between the visual and auditory conditions (Range ML: *p* = 1.000; SD ML: *p* = 0.329).

No significant main effects of cognitive conditions were observed for velocity or any direction-specific parameters in the AP direction (Table 1; Figure 2B,D).

### 3.2. Spectral Analysis

**Figure 3:**
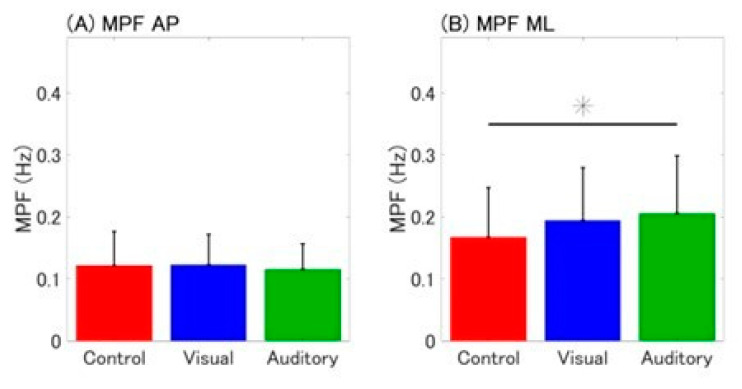
Bar plot of mean power frequency (MPF) for each cognitive condition. Mean and standard deviation (SD) of (**A**) MPF in the anteroposterior (AP) direction and (**B**) MPF in the mediolateral (ML) direction are presented. Error bars indicate standard deviations. * *p* < 0.05.

A significant main effect of cognitive conditions was observed for MPF ML but not for MPF AP (Table 2; Figure 3). Post hoc analysis revealed that MPF ML was significantly higher under the auditory condition compared to the control condition (*p* = 0.038; Figure 3B). However, no significant differences were observed between the control and visual conditions or between the visual and auditory conditions (control vs. visual: *p* = 0.118; visual vs. auditory: *p* = 1.000).

**Figure 4:**
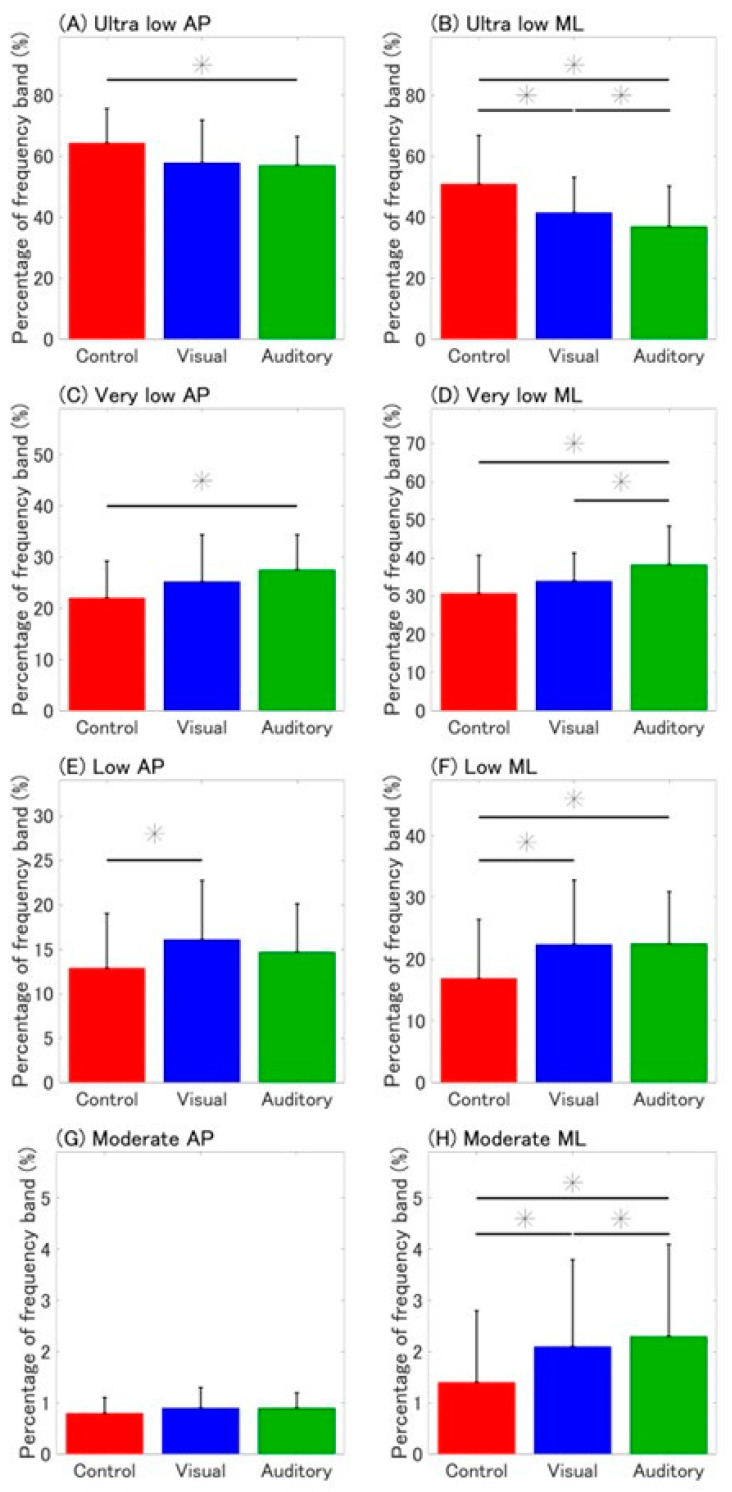
Bar plot of power spectrum density under each cognitive condition. Mean and standard deviation (SD) of (**A**,**B**) ultra-low frequency band in the anteroposterior (AP) and mediolateral (ML) directions, (**C**,**D**) very low frequency band in the AP and ML directions, (**E**,**F**) low frequency band in the AP and ML directions, and (**G**,**H**) moderate frequency band in the AP and ML directions are presented. Error bars indicate standard deviations. * *p* < 0.05.

Furthermore, rANOVA revealed a significant main effect of cognitive conditions for power spectrum density across all frequency bands in both directions, except for the moderate frequency band in the AP direction (Table 2). In the AP direction, the ultra-low frequency band significantly decreased under the auditory condition compared to the control condition (*p* = 0.006; Figure 4A), but no significant differences were observed between the control and visual conditions or between the visual and auditory conditions (control vs. visual: *p* = 0.070; visual vs. auditory: *p* = 1.000). Additionally, the power spectrum density of the very low frequency band significantly increased under the auditory condition compared to the control condition (*p* = 0.009; Figure 4C), but no significant differences were observed between the control and visual conditions or between the visual and auditory conditions (control vs. visual: *p* = 0.399; visual vs. auditory: *p* = 0.850). The power spectrum density of the low frequency band was significantly larger under the visual condition compared to the control condition (*p* = 0.011; Figure 4E), but no significant differences were observed between the control and auditory conditions or between the visual and auditory conditions (control vs. auditory: *p* = 0.277; visual vs. auditory: *p* = 0.597). In the ML direction, the ultra-low frequency band was significantly smaller under the auditory condition than the control and visual conditions (control: *p* < 0.001; visual: *p* = 0.035; Figure 4B). Additionally, the spectrum density under the visual condition was significantly smaller than under the control condition (*p* = 0.003). Conversely, the very low frequency band under the auditory condition was significantly larger compared to both the control and visual conditions (control: *p* = 0.002; visual: *p* = 0.017; Figure 4D), while no significant differences were observed between the control and visual conditions (*p* = 0.474). The low frequency band significantly increased under both the visual and auditory conditions compared to the control condition (*p* = 0.005 and *p* < 0.001, respectively; Figure 4F), with no significant difference observed between the visual and auditory conditions (*p* = 1.000). Similarly, for the moderate frequency band, spectrum density significantly increased under the auditory condition compared to both the control and visual conditions (control: *p* < 0.001; visual: *p* = 0.041; Figure 4H). Additionally, spectrum density for the moderate frequency band under the visual condition was significantly larger than that under the control condition (*p* < 0.001).

### 3.3. Cognitive Performance

A significantly lower correct response rate was observed in the auditory condition compared to the visual condition (auditory: 91.9 ± 10.3%, visual: 97.9 ± 3.4%; *p* < 0.001)

## 4. Discussion

To the best of our knowledge, this study is the first to compare the differential effects of cognitive tasks on static postural control attributable to sensory modalities while controlling for differences in cognitive task content. As anticipated, participants exhibited a smaller sway area, Range ML, and SD ML under both the visual and auditory conditions compared to the control condition. However, MPF ML was higher under the auditory condition than under the control condition, but not under the visual condition. Additionally, the spectrum density of the moderate frequency band in the ML direction was larger under the auditory condition than under both control and visual conditions. These findings suggest that cognitive tasks involving different sensory modalities can influence postural control during a quiet stance, even when the task content remains the same.

### 4.1. Effects by Cognitive Loads on Static Postural Control

Under both visual and auditory conditions, participants exhibited smaller sway areas and amplitudes in the ML direction than in the control condition. These findings suggest that motor-cognitive dual-task conditions can reduce postural sway during a quiet stance. Furthermore, our non-linear analysis revealed that the motor-cognitive dual-task condition elicited larger contributions not only to the low frequency bands, but also to the moderate frequency compared to the single motor task condition (control condition). Increased contributions to the low frequency bands are associated with a shift toward automatic postural control, as previously reported [29,30]. Conversely, other studies indicate that high contributions to the moderate frequency band reflect increased muscular activity, signifying a stiffness strategy [27,31]. Additionally, Zaback et al. [40] reported that muscle co-contraction and contributions to the moderate frequency band decrease when automatic postural control is dominant. Therefore, these observations suggest that cognitive load during a quiet stance may provoke a stiffness strategy rather than automatic postural control. In other words, although cognitive load reduces postural sway, it does not necessarily enhance postural control. Instead, it may lead to inefficient postural control, characterized by reduced redundancy [23,26].

However, our findings contrast with previous studies suggesting that cognitive tasks facilitate automatic postural control [12,13,24,29]. This discrepancy may stem from differences in task conditions that prioritize cognitive performance. Previous studies did not record cognitive accuracy or instruct participants to prioritize accuracy. In our study, participants were explicitly instructed to respond in the cognitive tasks as accurately as possible, achieving approximately 90% accuracy. This emphasis may have compelled participants to prioritize cognitive tasks over postural control, thereby increasing the cognitive load’s influence on postural stability. Therefore, future studies should investigate whether differences in instructions that prioritize postural control versus cognitive tasks lead to changes in the impact of cognitive load on static postural control.

Unlike the postural sway in the ML direction, no significant changes in postural stability measurements were observed in the AP direction across the three conditions. This directional specificity may be attributed to the experimental setup. In the visual condition, participants performed the PVSAT, while in the other two conditions, they gazed at a designated black point in the center of the screen. Previous research has demonstrated that postural control in the ML direction is influenced by the central field of view, whereas postural control in the AP direction is influenced by the peripheral field of view [41,42,43]. Consequently, focusing on the center of the screen may have heightened participants’ susceptibility to cognitive loads within the central field of view, predominantly affecting postural control in the ML direction.

These findings suggest that cognitive tasks may influence postural control in the ML direction and provoke a stiffness strategy regardless of sensory modalities. Interestingly, postural control in the ML direction strongly correlates with fall risk [19,44]. Therefore, our results imply that motor-cognitive dual-task conditions may serve as an effective method for assessing future fall risks.

### 4.2. Differential Effects on Postural Control by Sensory Modality

In the ML direction, participants exhibited a significantly larger contribution to the moderate frequency band under the auditory condition compared to the visual condition. Additionally, in the AP direction, visual cognitive loads induced larger contributions to the low frequency band, whereas auditory cognitive tasks elicited larger contributions to the very low frequency band. These findings suggest that cognitive tasks involving differential sensory modalities can alter postural control during a quiet stance, even when the cognitive task content remains the same.

In terms of the ML direction, participants demonstrated smaller sway amplitude and variability alongside higher MPF under the auditory condition but not under the visual condition. Previous studies have associated increased MPF accompanied by smaller sway amplitude or variability with a shift toward a stiffness strategy [23,45,46]. Therefore, our findings suggest that auditory cognitive tasks are more likely to provoke a stiffness strategy during a quiet stance compared to visual cognitive tasks. In addition to influencing postural control strategies, auditory cognitive tasks may also induce sensory reweighting. Specifically, our results showed decreased power spectrum density of the ultra-low frequency band and increased power spectrum density of the very low frequency band during auditory cognitive tasks. Previous studies have linked the ultra-low band to visual system contributions and the very low band to vestibular system contributions [27,28,47,48]. During additional cognitive tasks performed while standing, the visual system may prioritize the cognitive task over postural control, leading to reduced contributions to the ultra-low frequency band [49]. Simultaneously, vestibular inputs may play a compensatory role by responding to postural sway in the AP direction [50]. Thus, our findings suggest that auditory cognitive tasks facilitate reweighting from visual to vestibular inputs to maintain postural stability.

Overall, these results indicate that auditory cognitive tasks exert a greater influence on postural control during a quiet stance than visual cognitive tasks. One explanation for this sensory specificity may lie in cognitive involvement. Auditory feedback reportedly demands greater cognitive involvement compared to visual feedback [51,52,53]. Consequently, auditory cognitive tasks may require more attentional capacity, reducing the capacity available to maintain postural stability. This limitation in attentional resources may prompt a shift toward a stiffness strategy, which, although less redundant, effectively stabilizes postural balance.

Contrary to the findings from non-linear analysis, there was no significant difference in linear measurements of postural stability between the visual and auditory conditions. Visual cognitive tasks reportedly decrease postural sway more than auditory cognitive tasks [35,36]; however, this was not supported by our findings. These discrepancies may be explained by differences in participant demographics, as participants in previous studies were older adults, unlike the young adults in our cohort. Indeed, as per previous research, cognitive tasks reduce postural sway in older adults but not in young adults [54]. Older adults typically exhibit poorer postural control abilities and cognitive function, making them more susceptible to cognitive interference. As a result, they are more likely to prioritize postural control during motor-cognitive dual tasks. Participants in our study exhibited approximately half the sway area in both the visual and auditory conditions compared to those in prior studies, which reported sway areas of about 500–600 mm^2^ regardless of age [35,36]. Additionally, our participants achieved a correcting rate exceeding 90%, whereas participants in previous studies achieved correcting rates below 85%. These observations suggest that our participants had superior postural control abilities and cognitive function. Consequently, regardless of whether visual or auditory cognitive tasks were performed, participants’ postural sway may have reached a floor effect. Another potential explanation for these differences is the difficulty level of the cognitive tasks. In fact, more difficult cognitive tasks exert a greater influence on postural control [34,55]. Consistent with this, our findings demonstrated that the more challenging PASAT [37,56] had a greater influence on postural strategies.

These observations suggest that the effects of cognitive tasks on postural control may depend on the sensory modalities, and the auditory cognitive task had a greater influence on postural control compared to the visual cognitive task. Future studies should investigate the influence of cognitive tasks on postural control under dual-task conditions, focusing on different sensory modalities while controlling for both content and difficulty levels of cognitive tasks.

### 4.3. Limitations and Suggestions for Future Studies

This study has two limitations. First, the study was conducted with a small cohort of healthy young participants. The average correct response rate exceeded 90% in both the visual and auditory conditions, suggesting a ceiling effect [57]. Consequently, our cohort may have underestimated the influence of cognitive loads on postural control. In addition, previous studies reported that older adults or people with a history of falls were susceptible to cognitive interference [54,58]. Furthermore, people with cognitive impairments showed a reduction in dual-task function [59]. Therefore, it is necessary to recruit older adults, or individuals with a history of falls or cognitive impairments, in order to understand better the effects of sensory modalities in cognitive tasks on postural control. Second, we did not collect electromyography (EMG) data. Our findings indicate that cognitive load causes a stiffness strategy rather than automatic postural control based on the results of non-linear analysis. However, previous studies suggested that a shift toward automatic postural control due to cognitive tasks did not involve increased muscular activities [13,15,60]. Therefore, to gain a better understanding of postural control mechanisms, future studies should include direct measurements of muscular activity during motor-cognitive dual tasks.

## 5. Conclusions

This study examined the effects of cognitive tasks on static postural control across different sensory modalities while controlling for differences in task content. Our findings suggest that both visual and auditory cognitive tasks induce a stiffness strategy during a quiet stance. However, auditory cognitive loads exerted a greater influence on postural control than visual cognitive loads during motor-cognitive dual tasks. These results provide valuable insights into how cognitive loads impact postural control and highlight the sensory modalities that may be most effective for fall-risk assessments. Future studies should explore the effects of sensory modality-specific cognitive loads in older adults, individuals with a history of falls, or those with cognitive dysfunction. Additionally, research should investigate interventions that can mitigate the effects of cognitive loads on postural control during motor-cognitive dual tasks.

## Figures and Tables

**Table 1:** Mean and standard deviations (SD) of postural stability measurements under each cognitive condition.

	Control		Visual		Auditory		ANOVA	
	Mean	SD	Mean	SD	Mean	SD	*p*-Value	*η* ^2^
**Sway area (mm^2^)**	**434.7**	**234.5**	**317.6**	**191.0**	**316.7**	**182.3**	**0.001**	**0.280**
Range AP (mm)	23.2	6.6	21.0	6.7	22.7	8.1	0.228	0.065
**Range ML (mm)**	**26.2**	**7.8**	**22.0**	**5.6**	**21.5**	**4.6**	**<0.001**	**0.481**
Velocity AP (mm/s)	6.8	1.8	6.7	1.8	7.0	2.0	0.273	0.057
Velocity ML (mm/s)	8.5	2.4	8.7	2.5	8.8	2.4	0.14	0.086
SD AP (mm)	4.6	1.5	4.1	1.5	4.3	1.6	0.162	0.079
**SD ML (mm)**	**4.9**	**1.6**	**4.0**	**1.1**	**3.8**	**0.9**	**<0.001**	**0.505**

Cognitive conditions compared using rANOVA. Values in bold indicate a significant main effect at *p* < 0.05. rANOVA, repeated-measure analysis of variance; AP, anterior-posterior; ML, mediolateral.

**Table 2:** Mean and standard deviations (SD) of the mean power frequency (MPF) and power spectrum density of the four frequency bands under each cognitive condition.

	Control		Visual		Auditory		ANOVA	
	Mean	SD	Mean	SD	Mean	SD	*p*-Value	*η* ^2^
AP direction								
MPF (Hz)	0.122	0.058	0.123	0.048	0.116	0.043	0.074	0.012
**Ultra-low (%)**	**64.3**	**11.6**	**57.8**	**14.3**	**57.0**	**9.7**	**0.007**	**0.202**
**Very low (%)**	**22.0**	**7.4**	**25.2**	**9.4**	**27.5**	**7.0**	**0.024**	**0.156**
**Low (%)**	**12.9**	**6.2**	**16.1**	**6.7**	**14.7**	**5.5**	**0.012**	**0.183**
Moderate (%)	0.8	0.3	0.9	0.4	0.8	0.3	0.132	0.088
ML direction								
**MPF (Hz)**	**0.168**	**0.082**	**0.195**	**0.086**	**0.206**	**0.096**	**0.024**	**0.157**
**Ultra-low (%)**	**50.9**	**16.2**	**41.5**	**11.7**	**37.0**	**13.5**	**<0.001**	**0.473**
**Very low (%)**	**30.7**	**10.2**	**34.0**	**7.4**	**38.2**	**10.4**	**0.003**	**0.269**
**Low (%)**	**16.9**	**9.7**	**22.4**	**10.7**	**22.5**	**8.5**	**<0.001**	**0.358**
**Moderate (%)**	**1.4**	**1.5**	**2.1**	**1.8**	**2.3**	**1.8**	**<0.001**	**0.624**

Cognitive conditions compared using rANOVA. Values in bold indicate significant main effect at *p* < 0.05. rANOVA, repeated-measure analysis of variance; MPF, mean power frequency; AP, anterior-posterior; ML, mediolateral.

## Data Availability

The data presented in this study are available on request from the corresponding author.
